# A model for relative biological effectiveness of therapeutic proton beams based on a global fit of cell survival data

**DOI:** 10.1038/s41598-017-08622-6

**Published:** 2017-08-21

**Authors:** Ramin Abolfath, Christopher R. Peeler, Mark Newpower, Lawrence Bronk, David Grosshans, Radhe Mohan

**Affiliations:** 1 Departments of Radiation Physics, The University of Texas MD Anderson Cancer Center, Houston, TX 77030 USA; 2 Radiation Oncology, The University of Texas MD Anderson Cancer Center, Houston, TX 77030 USA

## Abstract

We introduce an approach for global fitting of the recently published high-throughput and high accuracy clonogenic cell-survival data for therapeutic scanned proton beams. Our fitting procedure accounts for the correlation between the cell-survival, the absorbed (physical) dose and the proton linear energy transfer (LET). The fitting polynomials and constraints have been constructed upon generalization of the microdosimetric kinetic model (gMKM) adapted to account for the low energy and high lineal-energy spectrum of the beam where the current radiobiological models may underestimate the reported relative biological effectiveness (RBE). The parameters (*α*, *β*) of the linear-quadratic (LQ) model calculated by the presented method reveal a smooth transition from low to high LETs which is an advantage of the current method over methods previously employed to fit the same clonogenic data. Finally, the presented approach provides insight into underlying microscopic mechanisms which, with future study, may help to elucidate radiobiological responses along the Bragg curve and resolve discrepancies between experimental data and current RBE models.

## Introduction

One of the main advantages of using protons and other ions in radiation therapy is the Bragg peak and its sharp distal dose gradient. This enables high dose delivery to deep seated tumors^1^.

While biological optimization is used for heavy ion therapy, for proton therapy a constant relative biological effectiveness (RBE) value of 1.1 continues to be employed clinically, despite experimental evidence that indicates a non-static RBE. A complete and robust model describing the cellular response and the biological effectiveness of protons along the Bragg curve would facilitate the biologic optimization of proton plans. However, uncertainties in the biological data at locations at or just distal to the Bragg-peak, where the charged particle LET is the highest, represent a particular challenge for the biological measurements and dose calculation in treatment planning systems^2–6^.

In general, RBE depends on the radiation dose, the fractionation scheme, the biological endpoint, the radiation quality, dose rate, and the tissue and cell-type. Radiation quality includes the type of radiation, the energy and the linear energy transfer (LET) that describes the energy deposited from the beam into the irradiated material, per unit particle path length (in units of *keV*/*μm*). As a charged particle penetrates tissue, it slows down and its LET increases. Experimentally RBE reaches a maximum at a certain depth and then decreases^7^.

A constant RBE of 1.1 has been recommended for clinical proton therapy^8–12^, despite the fact that variable RBE values have long been found experimentally^13–17^. *In vitro* experimental data reveal that the RBE depends on dose and the proton energy spectrum at the point of measurement^13, 14^. On the other hand, *in vivo* biological responses present a more complex expression showing smaller variations in RBE values for shallow depths. More recent studies have shown a significant increase of RBE at the end of the proton range^15–18^.

Given these laboratory results, it is likely necessary to develop a method to predict the biological effects of proton beams for given physical and geometric characteristics. One of the first attempts at modeling RBE based on track structure theory of heavy ions and neutrons was proposed by Butts and Kraft^19–21^. Implementation of such models in clinical applications requires physical input parameters such as, local particle energy spectrum and, biological input parameters, such as cell nuclear size, end damage response, etc. Currently, access to such parameters is lacking due to the computational complexities required to obtain them.

Scholz, Krämer, and Kraft^22–25^ implemented track structure models into particle radiation therapy that led to a model known as local effect model (LEM). Subsequently Paganetti^26^ studied amorphous track structure models in proton radiation therapy and showed that the model needed a critical adjustment of the parameters to improve prediction. Recently, Elsasser *et al*.^27^ proposed a modified local effect model which correlated spatial radiation damage and achieved good agreement with experimental data for different cell lines and several particle types, namely for protons and carbon ions.

First principle approaches have also been tried in many studies^28–32^. Neary discussed a linear dependence of chromosome aberrations with LET^31^. Hawkins derived a complex exponential relationship between *α* and lineal-energy and LET using microdosimetric kinetic modeling for several cell lines^28, 29^. The Wilkens and Oelfke linear model^30^ predicted the experimental data for LET values up to 30 *keV*/*μm* ^30^ well but was unable to predict the decrease in RBE with further increases of LET. Subsequent refinements of this model were reported in refs 33, 34. The repair-misrepair fixation (RMF) model of chromosome aberration that is one of the bases of our present model and computational analysis was developed in refs 35–37.

The Particle Irradiation Data Ensemble (PIDE) is an effort by Gesellschaft für Schwerionenforschung (GSI) to organize hundreds of radiation biology experiments in one dataset to facilitate evaluation of RBE models^38^. Based on their analysis of over 800 cell survival experiments, they conclude that RBE depends on particle species and the *α*/*β* ratio and that using LET alone is insufficient to predict RBE. In agreement with this assessment, the experimental data gathered in PIDE is a good collection to examine the fitting method introduced in the current study. We postpone to present this study to our future works and publications. In addition, Mohan *et al*.^39^ recently provided a comparison of the RBE data from Guan *et al*. for H460 non-small cell lung cancer cells to the RBE predicted by a number of models, including those from Wilkens and Oelfke^30^, Wedenberg *et al*.^34^, Carabe-Fernandez *et al*.^40^, McNamara *et al*.^41^, Chen and Ahmad^42^, Carlson *et al*.^35, 43^, and Frese *et al*.^37^. All of the models predicted lower RBE for the higher LET values used in the experiments. In this work we therefore focus on constructing a model for experimental data recently published by Guan *et al*.^32^ as existing models do not adequately explain these data.

Our goal is to provide a theoretical framework in resolving the discrepancies between such experimental data and theoretical models. As detailed in ref. 32, the design of a graded solid water compensator (jig) allows irradiation of cells by a mono-energetic scanning beam of protons at specific depths. The absorbed dose and the proton LET were calculated using the Geant4 Monte Carlo toolkit^32^. Subsequent high-throughput, automated clonogenic survival assays were performed to spatially map the biological effectiveness of scanned proton beams with high accuracy. This method aims to reduce uncertainties in the biological data.

In this study we developed an approach for global fitting of the recently published high-throughput and high accuracy clonogenic cell-survival data by Guan *et al*.^32^.

## Methods

We start by describing our phenomenological model, which is based on an extension of the microdosimetric kinetic model (MKM) originally proposed by Hawkins^28, 29^. We refer to our extension as generalized MKM (gMKM). In the following we elucidate the basis of gMKM and discuss in detail the differences with MKM.

In gMKM we first incorporate the effects of particle energy deposition spatial patterns calculated for single-tracks with nanometer spatial resolution. The initial energy of the proton beam and the geometry were consistent with the experimental beam line used in ref. 32. The proton energy ranges from 80 MeV to as low as 100 keV. The choice in low energy cut-off is based on the range of protons with 100 keV of energy, which is less than the diameter of a cell nucleus, e.g., 5 *μm* (typical diameter range is within 3–7 *μm* depending on several factors such as cell type, cell cycle, adherence, etc.). To this end, we used a combination of Geant4 and Geant4-DNA Monte Carlo toolkits (version 10.2) to simulate the track-structures in macroscopic and microscopic scales in water equivalent materials^44^.

We then analyzed the energy deposition distributions of protons and assessed if the Poisson distribution that is the central assumption in MKM effectively described the track structure. To this end, we randomly selected locations along the beam axis to score energy deposition in 5 *μm*-diameter spherical volumes representing cell nuclei. The dimensions of these volumes were consistent with the dimensions of the cell nucleus used in the clonogenic assays in ref. 32.

With these two steps, we systematically obtained the distribution of ionizations in geometrical structures resembling the cell nucleus as a function of their position relative to the source, proton energy, and LET along the radiation track.

**Figure 1:**
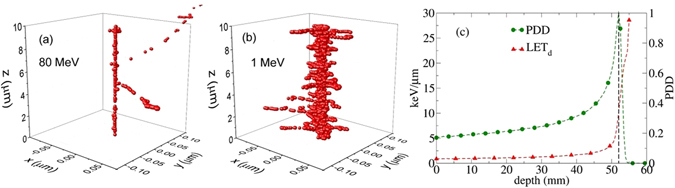
Two ionization-tracks of 80 MeV (**a**) and 1 MeV (b) protons obtained from the Geant4-DNA Monte Carlo simulation in water with initial points at x = y = z = 0 and directions parallel to z axis. The number of events in 1 MeV proton is one order of magnitude greater than 80 MeV. Such compact ionization events in low energies clearly justify a deviation from Poisson distribution that may describe the ionization in 80 MeV. The relative energy deposition along Bragg curve and LET for a beam of proton with 80 MeV is shown in (**c**). The line indicates the position of Bragg peak at approximate 5.2 cm depth corresponding with *LET *≈ 10 *keV*/*μm*.

**Figure 2:**
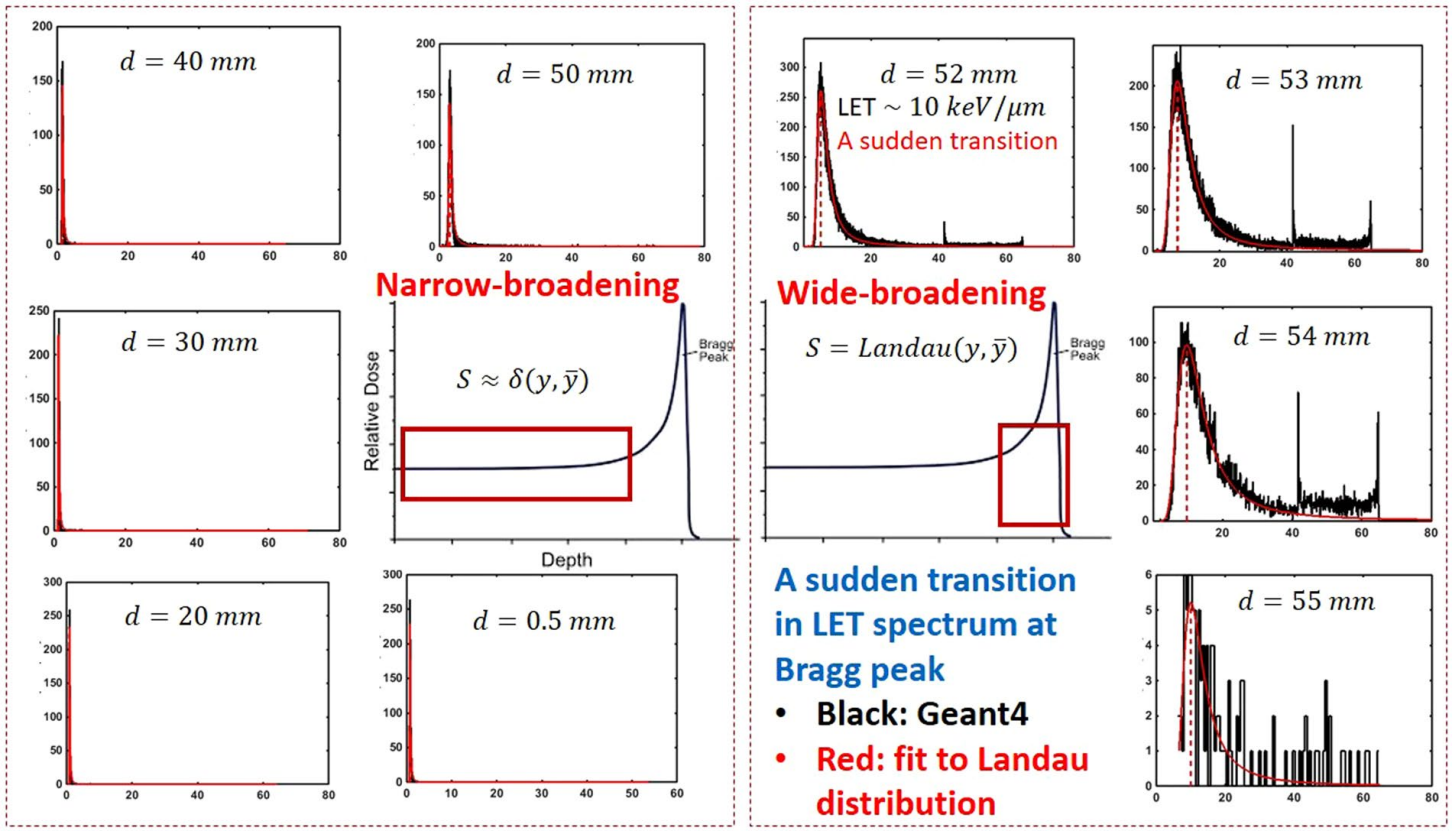
Series of the lineal energy spectrum and the fitting to Landau distribution is shown for a beam of proton with energy 80 MeV as an increasing function of depth in clockwise order. The x-axis and y-axis represent the lineal energy in *keV*/*μm* and the histogram of lineal energy in (*keV*/*μm*)^−1^. An abrupt transition in shape of the lineal energy distribution function from sharply symmetric to wide and asymmetric form is seen at 5.2 cm depth where *LET* ≈ 10 *keV*/*μm* (see Fig. 1c) and LET exceeds 10 *keV*/*μm*.

As shown in Fig. 1a, in locations near the beam entrance where the energy of the proton is relatively high, i.e. 80 MeV, and therefore the LET is low, the local density of ionization within the cell nucleus is sparse and can be fitted by a Gaussian and/or Poisson distribution. The spectrum of energy loss and lineal-energies as a function of depth are plotted in Fig. 2. The Gaussian/Poissonian approximation of energy-loss spectral density holds for a wide range of depths, from the entrance to locations proximal to the Bragg peak where the protons slow down to energies of approximately 15 MeV and LET ≈ 10 *keV*/*μm*. This result can be seen in Fig. 1c where the calculated energy deposition and LET as a function of depth using Geant4 for a circular shape source of proton consists of a Gaussian energy broadening.

Beyond *LET* ≈ 10 *keV*/*μm*, as shown in Fig. 1b, the atomistic excitations and ionizations undergo a transition to a highly compact track structure. This transition in energy loss spectrum to a clearly visible Landau distribution^45, 46^ occurs around *d* = 52 *mm* for the 80 MeV beam as shown in Fig. 2. Therefore, above *LET* ≈ 10 *keV*/*μm*, the deviation from a Poisson distribution appears to be significant.

In the Appendix we develop an analytical model based on Neyman’s distribution of type A (a compound Poisson distribution)^47, 48^ for illustration of the effects due to deviations from a Poisson distribution. In the following sections, however, we present numerical results of more sophisticated Monte Carlo calculations. In particular in Fig. 2, we present a series of lineal energy spectra, fitted to the Landau distribution^45, 46^ for a beam of protons with 80 MeV of energy. The spectrum evolves as an increasing function of depth (depicted in clockwise order). An abrupt transition in the lineal energy spectrum is seen at 5.2 cm depth where the energy deposition coincides with the Bragg peak (see Fig. 1c) and *LET *≈ 10 *keV*/*μm*. The transition in the lineal energy spectrum corresponds to a similar transition in biological responses. As previously demonstrated, over a wide range of depths (highlighted by a square in the energy deposition curve in the left) LET is low and the spectrum can be approximated by a sharp and symmetric Gaussian/Poissonian distribution. At high LET’s the Gaussian symmetry in lineal-energy distribution around its statistical mean value gradually breaks down. According to this analysis, the non-linearity in RBE stems from this symmetry breaking in the lineal energy spectrum. Moreover, we attribute a decrease in RBE beyond very high LET’s, a phenomenon known as the over-kill effect in the literature^7^, to the widening and sparsity of the lineal energy distribution (see for example the spectrum at *d* = 55 mm) and lack of beam strength due to a sharp drop in total energy deposition and particle fluence. These observations have been transpired to our practical approach in the fitting procedure where two kinds of linear and non-linear polynomials were converged and fitted to the cell survival data below and beyond *LET* ≈ 10 *keV*/*μm*.

We now turn to highlight the mathematical framework we employed in fitting the experimental data using LET power series in *α* and *β*. We refer the interested readers to the mathematical details in the Appendix.

The results obtained from experiments^32^ as well as simulations^37^ confirm that as LET increases, *α* and *β* evolve from linear to non-linear functions of LET. Similar to MKM, in gMKM we calculated the cell survival-fraction by integrating over the solutions of the repair-misrepair-fixation (RMF) model^35, 49, 50^. The RMF model links radiation double strand break (DSB) induction and the processes that lead to exchange-type chromosome aberrations and cell death. The linear-quadratic (LQ) survival model has been shown to be an approximate time-integrated solution to the RMF model that accounts for incorrect chromosome end joining. More accurate consideration of the RMF model by perturbative linearization of the non-linear solutions leads to the cell survival-fraction, *SF*, represented as a power series of the statistical moments of the specific energy *z* in a cell-nucleus domain

1 $$-\mathrm{ln}(SF)={a}_{1}\bar{z}+{a}_{2}\overline{{z}^{2}}+{a}_{3}\overline{{z}^{3}}+\cdots .$$

Note that the higher order terms beyond the MKM LQ-model in Eq. (1) are from a power expansion of RMF solutions. Such non-linearity in SF have been examined before to analyze lack of symmetry in split-dose survival of mouse jejunal crypt cells where total dose was fixed but asymmetric combinations of the dose were used, i.e., large following small vs. small following large dose fractions^51^. In Eq. (1), ${a}_{1},\,{a}_{2},\,\ldots$ are expansion coefficients and $\bar{z},\,\overline{{z}^{2}},\ldots$ are the ensemble averaged moments of the specific energy. Note that ${a}_{1},\,{a}_{2},\ldots$ are combinations of the DNA-DSB induction strength constants in the RMF model and the pair-wise repair-misrepair chromosome end-joining rate coefficients.

To perform the averaging in Eq. (1) explicitly, the distribution function of these random variables must be known. In MKM, the Poisson distribution is assumed to govern the distribution of ionization events. Based on our analysis, this is a plausible assumption in the low LET limit, however, a correction to the Poisson distribution is warranted for high LETs. We therefore expand the non-Poissonian distribution function of ions in the vicinity of the Poisson distribution. Following algebraic manipulation, an expansion around the MKM mean specific energy ${\bar{z}}_{MKM}$ in Eq. (1) can be found. Using the macroscopic dose $D\equiv \bar{z}$ and dose-averaged lineal-energy *y*_*d*_ or equivalent *L* (simply LET that is a linear function of *y*_*d*_) as two independent variables in this equation, we find

2 $$-\mathrm{ln}(SF)=\sum _{i=1}^{n}\sum _{j=1}^{i}{b}_{i,j}{L}^{i-j}{D}^{j}.$$

As described in the Appendix, derivation of Eq. (2) involves expansion of moments $\overline{{z}^{2}},\overline{{z}^{3}},\ldots$ of a non-Poissonian distribution, in power series of Poissonian or MKM specific energies, ${\bar{z}}_{MKM}$, ${\bar{z}}_{MKM}^{2}$, ${\bar{z}}_{MKM}^{3}$, …, e.g., $\bar{z}={\sum }_{k=1}{c}_{k}{\overline{{z}^{k}}}_{MKM}$; $\overline{{z}^{2}}={\sum }_{k=2}{c}_{k}{\overline{{z}^{k}}}_{MKM}$; $\overline{{z}^{3}}={\sum }_{k=3}{c}_{k}{\overline{{z}^{k}}}_{MKM}$; … Thus the expansion coefficients *b*_*i*,*j*_ are linear combinations of ${a}_{1},\,{a}_{2},\ldots$ and ${c}_{1},\,{c}_{2},\,\ldots$.

Recalling *α* and *β* in the LQ cell-survival model, $-\mathrm{ln}(SF)=\alpha D+\beta {D}^{2}$, and truncating Eq. (2) up to the quadratic term in *D* gives

3 $$\alpha =\sum _{k=1}^{n}{b}_{k,1}{L}^{k-1},$$

and

4 $$\beta =\sum _{k=1}^{n-1}{b}_{k+1,2}{L}^{k-1},$$

with *n* ≥ 2. In Eqs (3 and 4) *α* and *β* are polynomials of LET with orders *n* − 1 and *n* − 2 respectively, thus the formula for *SF* contains 2*n* − 1 fitting parameters. For clarification of the compact notations used in Eqs (3 and 4), let us consider a class of polynomials truncated at *n* = 4, hence $\alpha ={b}_{1,1}+{b}_{2,1}L+{b}_{3,1}{L}^{2}+{b}_{4,1}{L}^{3}$ and $\beta ={b}_{2,2}+{b}_{3,2}L+{b}_{4,2}{L}^{2}$. A similar approach with slightly different representation of the RMF model as introduced by Carlson *et al*.^35^ provides a more elaborate interpretation of the polynomials in the above equations. According to our unpublished studies, $n=2,3,4,\,\ldots$ in *α* and *β* in Eqs (3 and 4) represent the levels of chromosomal aberration complexities such as the binary, ternary, quaternary, etc. chromosome recombination. We postpone the details of the higher order (beyond binary) chromosome recombination leading from Neyman’s distribution of type A^47, 48^ to our future publications.

In practice we aim to find an optimal *n* that allows the best fitting of the experimental data with minimal number of parameters. Thus, we need to calculate a minimal *n*. The lowest possible *n* in Eqs (3 and 4) corresponds to *n* = 2. This essentially corresponds to the linear *α* and constant *β* as function of LET in MKM. This model contains three fitting parameters.

We now turn to discuss the results of the fitting procedure for the clonogenic cell survival experimental data. We use global fitting of *SF* in a 3D parameter space using two independent variables *D* and *L*. Eq. (2) provides specific forms of polynomials that we may use to fit the data.

## Results and Discussion

In the current study we developed an approach for global fitting of the recently published high-throughput and high accuracy clonogenic cell-survival data.

A relation between cell survival and LET was extracted in a subsequent step after the calculation of the linear-quadratic cell survival parameters *α*, *β*, *α*/*β* and RBE. In ref. 32, and in all other RBE publications (to the best of our knowledge) the﻿﻿ fitting was performed for each individual survival curve with a specific average LET. This method of fitting individually fits cell survival curves with measured data for each LET value separately, which would have led to high degree of variability in alpha and beta parameters of the LQ model. As a result, the dependence of cell-survival data on LET exhibits fluctuations, and it would be challenging to fit and quantitatively interpret the results into an RBE model. Instead, we carry out a “global” fit with the measured data. This process reduces the overall uncertainty.﻿ In addition to this systematic uncertainty, there is another source of numerical noise in fitted data, largely stems from uncertainties in collecting survival data. In practice, the extraction of LET ﻿and its poistion dependence in﻿ cell-survival data may vary among different fitting and experimental procedures.

The present data in ref. 32 shows that the measured biologic effects are substantially greater than in most previous reports. It is characterized by a non-linear RBE as a function of LET near and beyond the Bragg peak. The calculated RBE is characterized by high sensitivity to small variations in LET distal to the Bragg peak, where a small uncertainty in the position of the cells may result in significant change in LET.

It is therefore crucial to search for appropriate fitting procedures and algorithms to be able to enhance the quality of the calculated RBE and the interpretation of the data. There are several models in the literature predicting regular and smooth dependence of RBE parameters on LET including the microdosimetric kinetic model (MKM), local effect model (LEM), and Monte Carlo damage simulation (MCDS). In this work, we explore more reliable fitting procedures which take into account a correlation among the SF curves. Our method is based on a three-parameter global fitting. We develop an optimization procedure that allows fitting a 2D surface in a 3D parameter space spanned by dose, LET and cell surviving fraction (SF).

An extension of this approach for particles heavier than protons is currently under investigation and the goal for such studies is to generate data needed to optimize treatment plans incorporating variable RBE.

**Figure 3:**
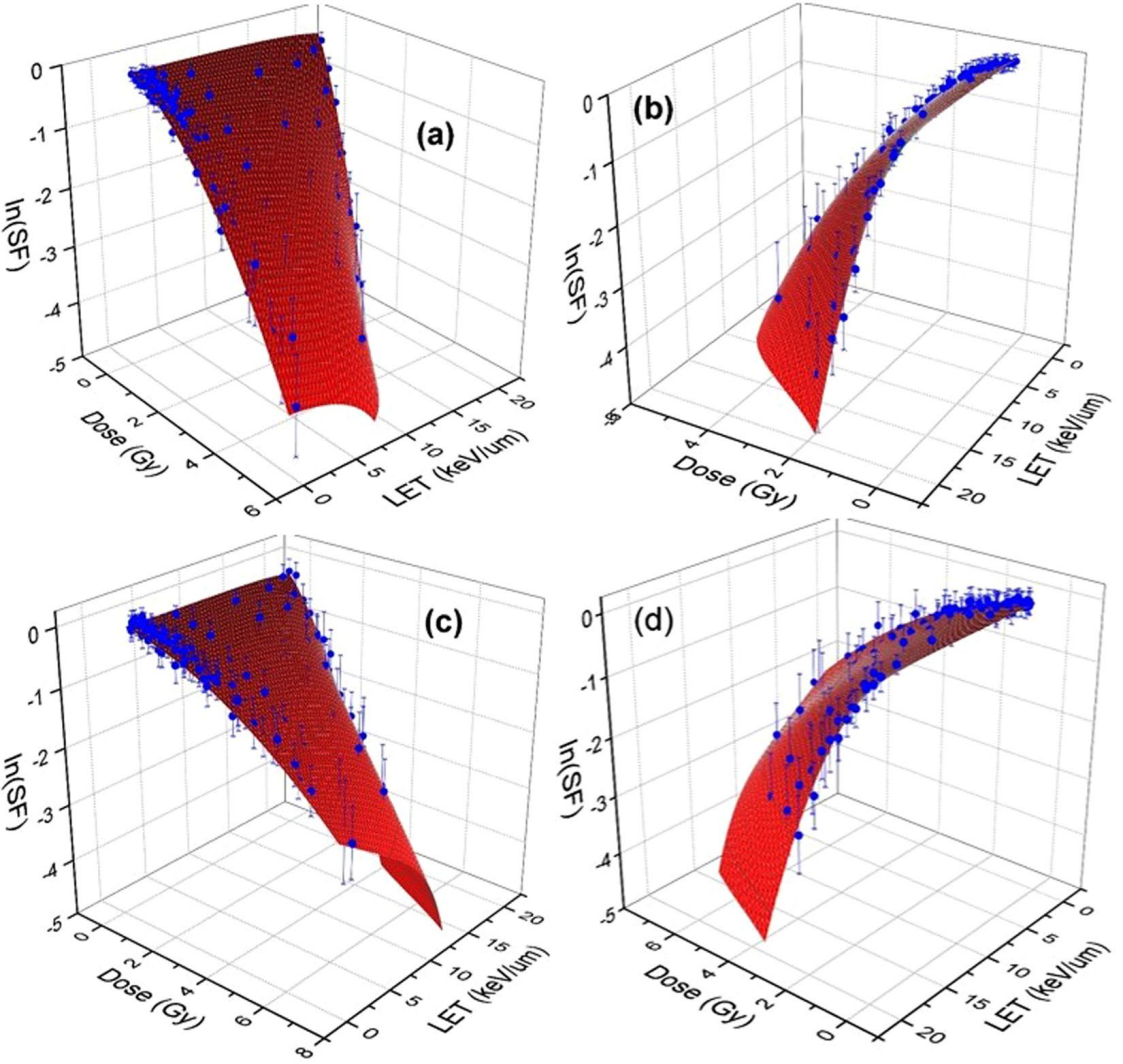
Experimental data of clonogenic survival fraction (SF) corresponding to H460 (**a**,**b**) and H1437 (**c**,**d**) cells are shown in blue dots with experimental error bars in logarithmic scale. The surfaces are fitted to the experimental data using global 3D optimization using a non-linear LET model as presented in the text. The left and right figures show the views from low and high LETs.

**Figure 4:**
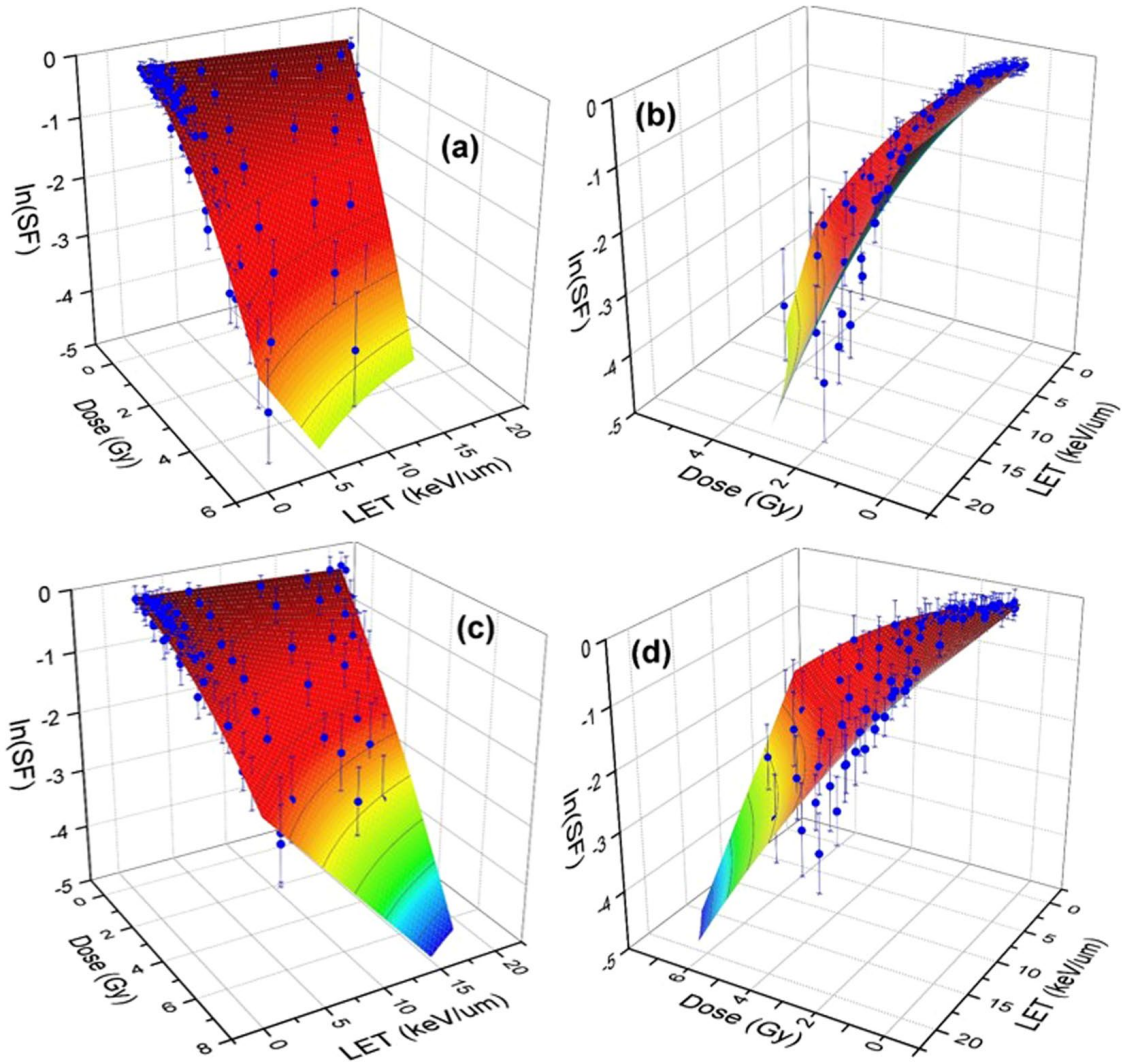
Shown same experimental data of clonogenic survival fraction (SF) for H460 (**a**,**b**) and H1437 (**c** and **d**) cells as in Fig. 3 with a difference that a linear LET model corresponding to *α* = *α*_0_ + *α*_1_ *L* and *β* = *β*_0_ have been used to obtain a globally optimal surface fitted to the experimental data. Similar to Fig. 3, the left and right figures show the views from low and high LETs.

A comparison between two schemes in global fitting using non-linear and linear polynomials are shown in Figs 3 and 4. In the linear polynomials fitting procedure, shown in Fig. 4, we considered $\alpha ={\alpha }_{0}+{\alpha }_{1}LET;\,\beta ={\beta }_{0}$ and obtained an optimal surface in the three-dimensions spanned by dose, LET and SF that fit the experimental points whereas in the nonlinear approach shown in Fig. 3, we used Eqs (3 and 4). Clearly the linear fitting procedure can not cover the high LET data points. This is in contrast with the non-linear fitting. The error bar shown in these figures is extracted from the experimentally reported values in ref. 32 and transformed to logarithmic scale using STD in log scale equivalent of STD/SF. Hence as we show the points with lower SF, the magnitude of error bars becomes more significant.

**Figure 5:**
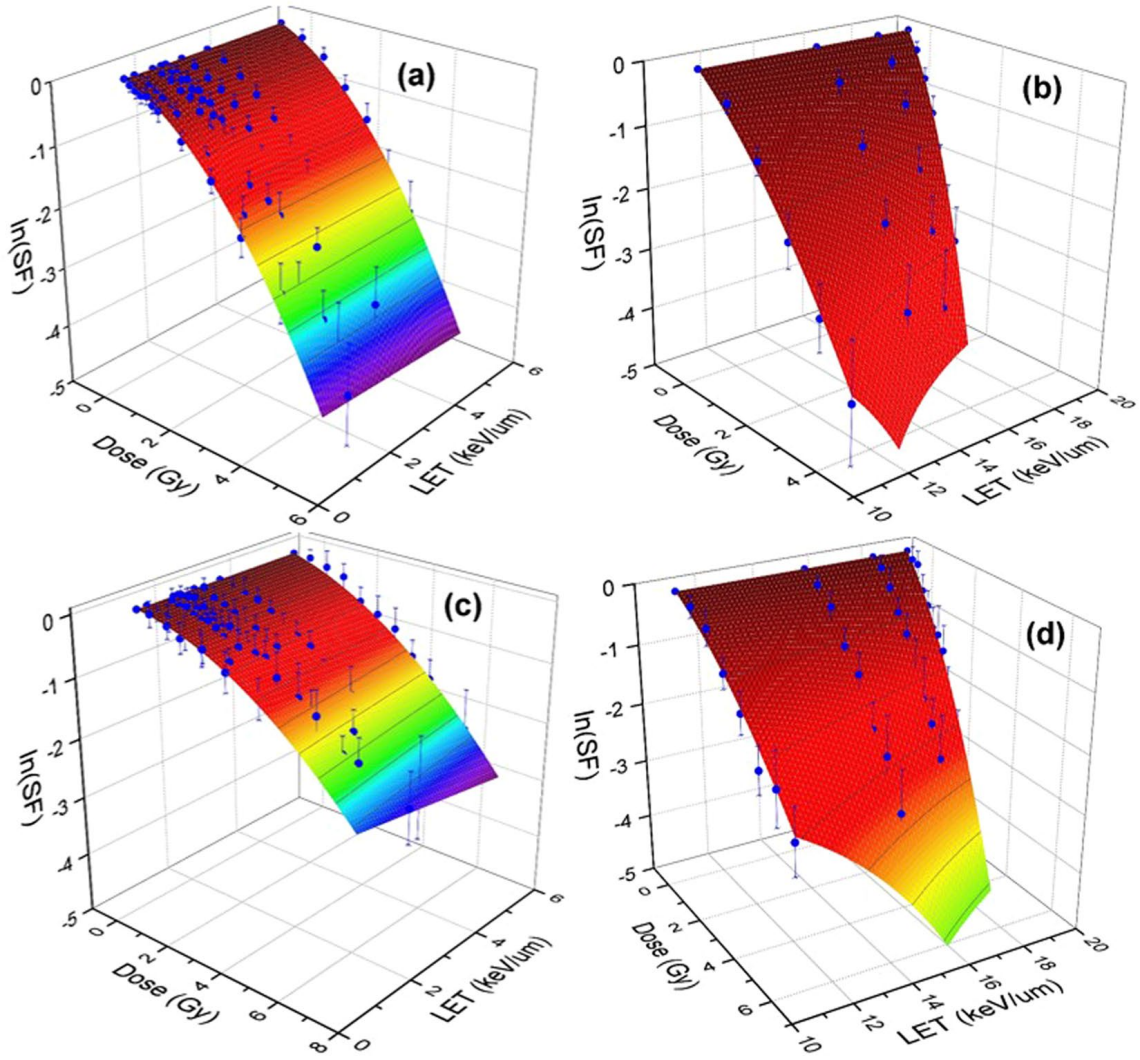
Shown H460 (**a**,**b**) and H1437 (**c**,**d**) cell survival surfaces in low (**a**,**c**) and high (b and d) LETs. As discussed in the text, *LET* ≈ 10 *keV*/*μm* separates the low LET from high LET domain for 80 MeV pencil beam of proton. The gMKM polynomials have been used in a 3D global fitting approach to obtain the best-fitted surface to the experimental data. The numerical values of the fitting parameters are in linear (**a**,**c**) and non-linear (**b**,**d**) domains are presented in Table 1.

In the non-linear fitting procedure we further divided LET into two domains of high and low as discussed in the Methods section. For low LET, a linear *α* and constant *β* which corresponds to choosing *n* = 2 in Eq. (2) can satisfactorily fit the data. The result of fitting for low LETs are shown in the left panel of Fig. 5(a,c).

For the completeness of our discussion, we reemphasize the expected transition from linear to non-linear biological responses along the energy deposition curve where LET increases monotonically. To characterize the threshold between “low” and “high” LETs, we calculated the lineal energy distribution function and used it as a metric to quantify the boundary between these two limits. In Fig. 2 we summarized the evolution of the lineal energy spectrum for a proton pencil beam with initial energy 80 MeV as a function of depth and LET in water. As discussed earlier, a transition from sharply peaked and approximately symmetric distribution in low LETs to highly asymmetric Landau-type distribution takes place. This transition happens in the vicinity of the Bragg peak where LET is approximately 10 *keV/μm* (see also Fig. 1c). We therefore consider this value of LET as a border in fitting the cell survival data that divides the domains of the low and high LETs for protons. Thus we consider *LET*≈10 *keV/μm* in the global fitting of cell survival as the value below which a linear RBE model is used whereas above *LET* ≈ 10 *keV/μm* a non-linear RBE model is implemented.

It is important to mention an abrupt transition in the lineal energy distribution may be attributed to a similar characteristic transition in the measurable biological quantities. For example the measured *α* and *β* are the result of superposition-convolution of the corresponding parameters with spectrum of lineal energies, i.e., $\alpha (L)={\int }_{0}^{\infty }\tilde{\alpha }(y)S(y)dy$, $\beta (L)={\int }_{0}^{\infty }\tilde{\beta }(y)S(y)dy$ where $L={\int }_{0}^{\infty }yS(y)dy/{\int }_{0}^{\infty }S(y)dy$, *S*(*y*) is the lineal energy distribution function, and $\tilde{\alpha }$ and $\tilde{\beta }$ are theoretically defined for a single value of lineal energy, *y*.

The numerical values of the fitting parameters, presented in Table 1, were calculated within an in house developed Matlab code designed for an optimization algorithm in searching the best 2D surface fitted to a data set in 3D space. An iterative procedure was employed to minimize the chi-square value to obtain the optimal parameter values and performing nonlinear curve fitting. The optimization core of our approach uses the implementation of Levenberg-Marquardt-Fletcher algorithm developed for nonlinear least squares fitting problems.

**Table 1:** Numerical values of expansion coefficients in *α* and *β* given in Eqs (3 and 4) for H460 and H1437 cell survivals.

	H460	H460	H1437	H1437
	Low LET	High LET	Low LET	High LET
*b*_1,1_	−0.21447	−0.12913	−0.05237	−0.12288
*b*_2,1_	−0.007	−6.733E-03	−0.0151	−0.00624
*b*_3,1_	0	−4.563E-04	0	−1.322E-06
*b*_4,1_	0	−3.37529E-05	0	−9.449E-07
*b*_5,1_	0	−8.16229E-06	0	−8.812E-07
*b*_6,1_	0	0	0	−7.725E-08
*b*_2,2_	−0.11061	−0.06864	−0.03265	−0.00382
*b*_3,2_	0	−0.00202	0	−0.0007821
*b*_4,2_	0	−1.36897E-04	0	−9.275E-05
*b*_5,2_	0	−3.63748E-05	0	−9.511E-07
*b*_6,2_	0	0	0	−1.68E-07
Reduced *χ* ^2^	0.00487	0.07557	0.00541	0.05472
*R* ^2^ (COD)	0.99496	0.98407	0.98233	0.96777
Adjusted *R* ^2^	0.99481	0.92833	0.98233	0.93911

For a beam proton with energy 80 MeV the transition between low and high LETs occurs at *LET* ≈ 10 *keV*/*μm*. The reduced chi-square, *χ* ^2^, the coefficients of determination (COD), *R* ^2^, and adjusted *R* ^2^ have presented to illustrate the goodness of the fitting procedure.

As seen in this table we obtain a satisfactory convergence with *n* = 5 and *n* = 6 for H460 and H1437 cell lines. The rationale for selecting large values of *n* stems from numerical methods in optimization and convergence of the self-consistent RBE solution in surface fitting procedure. The convergence of the power series can be seen from the numerical values presented in the table. By increasing the powers in the expansion, the numerical values of the coefficients will change, however, if the change in RBE resides within a convergence domain (chosen by the user) the optimization stops. Although in our approach we search for optimal surfaces to minimize chi-square values iteratively, it is known that additional metrics are needed to evaluate the goodness of the fit. We therefore report the calculated R-square, also known as coefficient of determination (COD) in Table 1. As shown in this table, the numerical values of R-square are close to 1. The closer the fit is to the data points, the closer R-square will be to the value of 1. It is also known that a larger value of R-square does not necessarily mean a better fit because of the degrees of freedom that can also affect the values. Thus we report the adjusted R-square values to account for the degrees of freedom.

To determine an optimal polynomial in the fitting process, we consider a series of polynomials corresponding to $n=2,3,4,\,{\rm{\ldots }}$ and perform optimization for each individual polynomial separately. A comparison based on chi-square and RBE is performed for polynomials with orders *n* to *n* + 1. If the difference between two chi-square’s (and RBE’s) in steps *n* and *n* + 1 is lower than a threshold value, e.g., $|{\chi }^{2}(n+1)-\,{\chi }^{2}(n)| < \delta$, the optimization stops and chi-square and RBE corresponding to step *n* + 1 is reported. In low LET’s this procedure converges to a linear polynomial (as the zeros in Table 1 shows the contribution of higher order power series are negligible).

To examine the stability of the converged solutions we consider an alternative approach in matching the low and high LET surfaces at the boundary where *LET* = 10 keV/*μ*m. To handle the discontinuity and obtain potentially smoother curves at the “transition point” where low LET data meet the high LET data, we include series of points below (and above) 10 keV/*μ*m to high (and low) LET and perform the fitting procedure and calculate the optimum high (low) LET surfaces. We then remove these points located in the vicinity of the boundaries and compare the resulting surfaces with a simple piece-wise matching of low and high LET data at the boundaries as shown in Figs 7, 8 and 9. Comparison between RBE’s, alpha’s and beta’s resulted in no significant difference.

**Figure 6:**
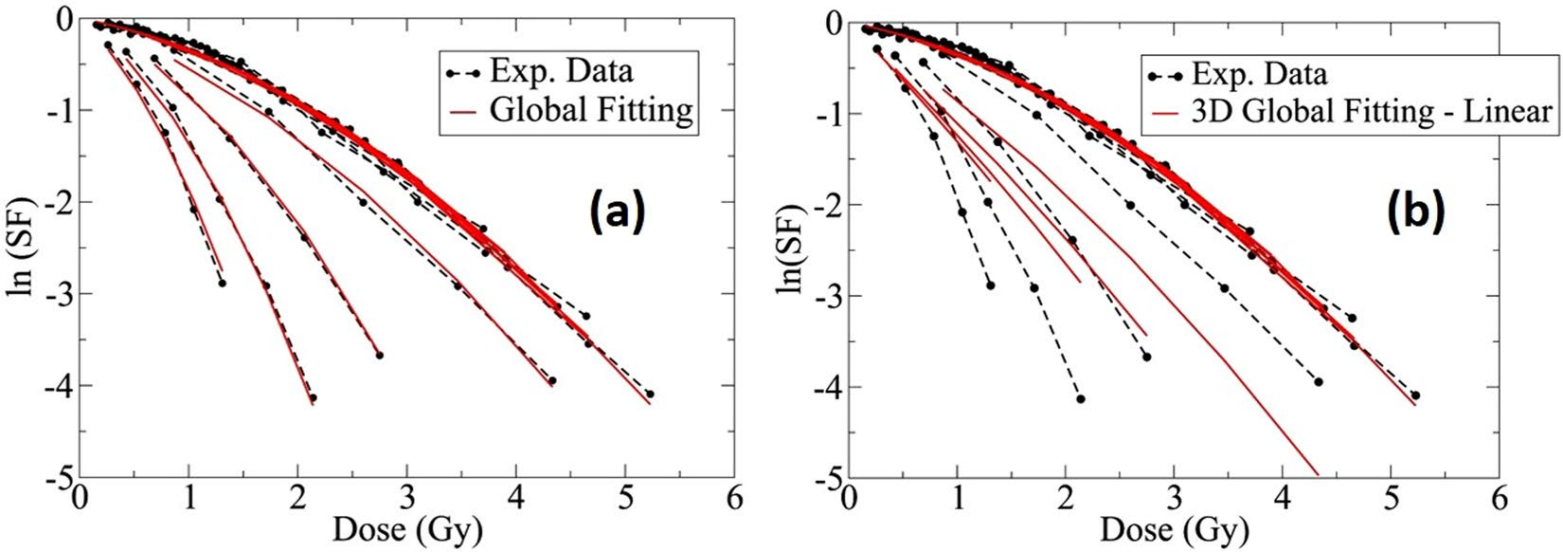
2D representation of the survival fraction shown in Fig. 3 (H460 cells) using non-linear gMKM fitting polynomials (left panel) and linear MKM polynomials in Fig. 4 (right panel). The black dotted lines represent the experimental data and the red lines are the fitted surfaces projected to the SF-Dose plane.

**Figure 7:**
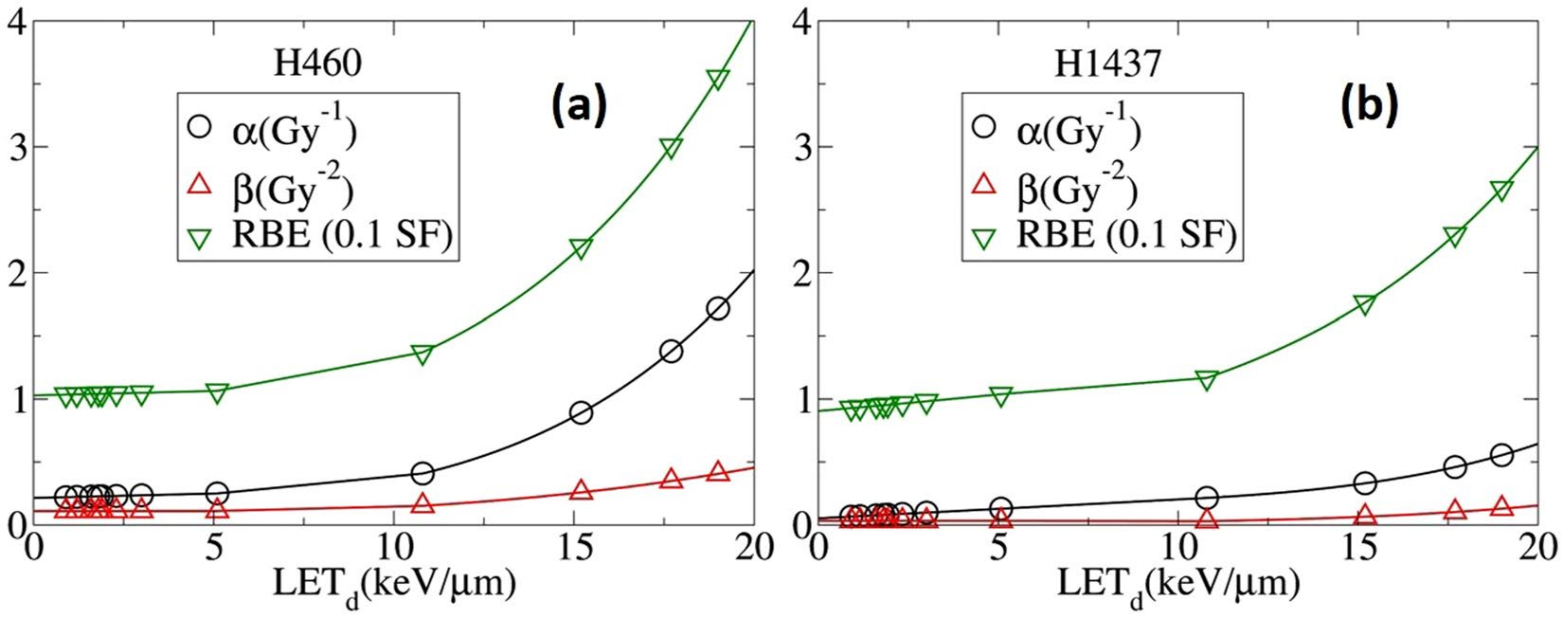
*α*, *β*, and RBE as a function of LET calculated analytically from *SF* equations obtained by the 3D global fitting of clonogenic cell-survival data as shown in Figs 2 and 3.

In Fig. 6 we show the same plots as in Fig. 5 except the 3D surface is projected onto the SF-dose plane. The first few curves from the top in Fig. 6 show the results of the *n* = 2 fitting for LETs less than $10\,keV/\mu m$. In Fig. 7, *β*, and RBE as a function of LET calculated from the fitted surfaces are plotted. For low LET, the linear dependence of *α*, *β*, and RBE is consistent with the fitting procedures introduced in other publications including Hawkins^28, 29^, Wilkens and Oelfke^30^ and Steinsträter *et al*.^52^ *α*.

Recently McNamara *et al*.^41^ fitted the present experimental data using a similar low-LET methodology^25^. The result of their calculation shows that present models may underestimate the high LET region of the LET-RBE relationship. This issue has been resolved within the presented methodology of gMKM. Using the fitting approach of the constructed framework, the last four red curves in Fig. 6 (left panel), as well as in Fig. 7, exhibit a reasonable fit to the high LET data. In this case, polynomials of order *n* = 6 re used for *α* and *β*.

In the right panel of Fig. 6 we show the result of similar 3D global fitting using linear MKM polynomials, e.g., $\alpha ={\alpha }_{0}+{\alpha }_{1}L$ and a constant *β*. As shown in this figure, the low LET data can be fitted well by a linear *α* and a constant *β*, same as the left panel of Fig. 6. However, for high LET the difference between the fitted curves and the experimental data is significant. Such difference can be reduced by using gMKM non-linear polynomials for *α* and *β* as we used for the fitting of high-LET data in the left panel for Fig. 6.

**Figure 8:**
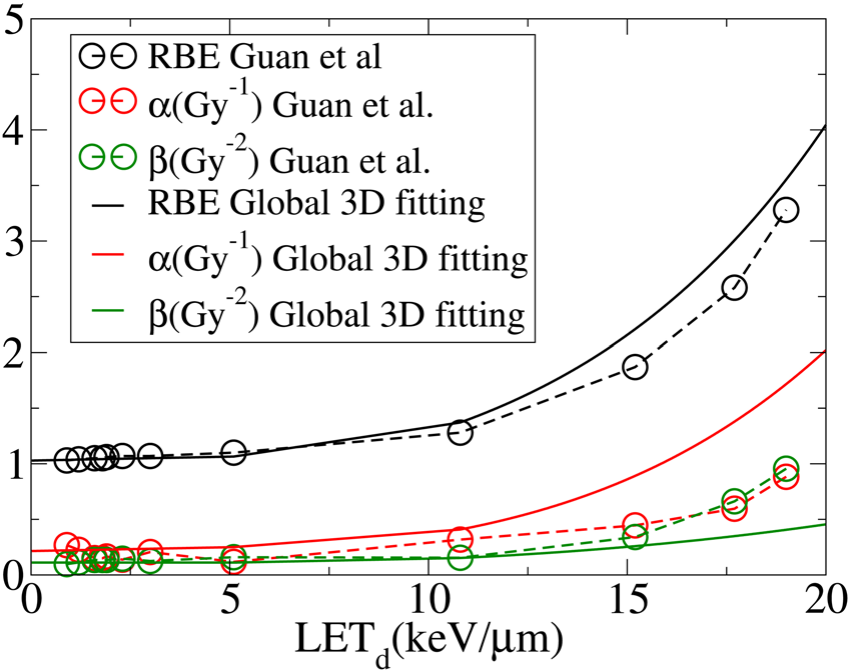
*α*, *β*, and RBE as a function of LET calculated by the conventional method and the presented 3D global fitting method. Although the difference in RBE between the two methods is negligible, the *α* and *β* are significantly different (H460 cells).

**Figure 9:**
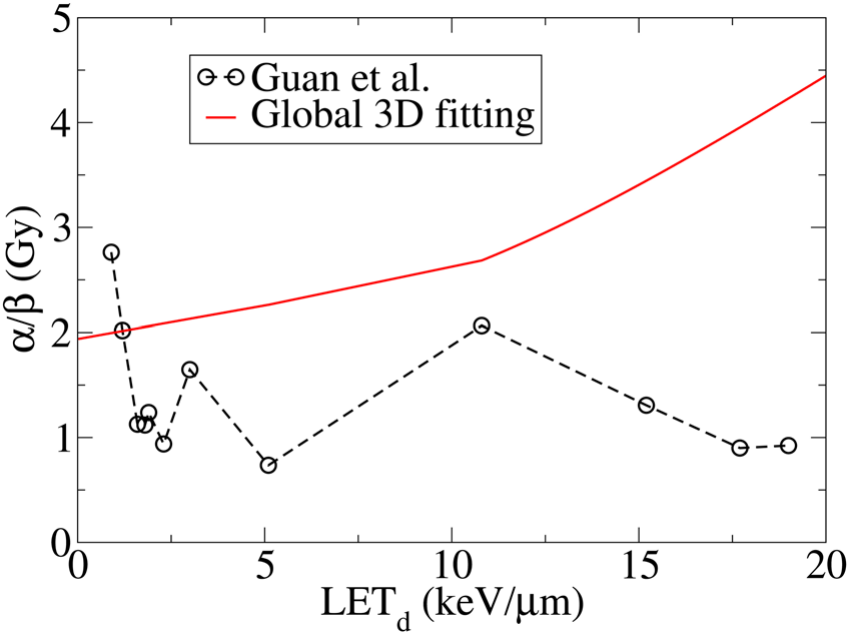
Lack of correlation among LETs in fitted cell-survival data in the conventional fitting procedure leads to strong fluctuations in *α*/*β* as reported by Guan *et al*.^32^. In contrast, *α*/*β* increases with increasing *LET*_*d*_ in 3D global fitting consistent with the cell inactivation target theory (H460 cells).

To illustrate the advantage of the present method, we compare the results from global 3D fitting with the conventional fitting procedure based on the same experimental data published recently by Guan *et al*.^32^. As mentioned before, in the conventional fitting procedure, each survival curve with a specific LET was fitted individually without considering correlations among them. Hence, it may lead to irregular forms of *α* and *β* as functions of LET. This method used to fit the data in ref. 32 is inconsistent with the experimental design that was intended to correlate the cell-survival data of different LETs along the Bragg curve. Figures 8 and 9 show comparison between the conventional and 3D global fitting methods.

The presented fitting approach prevents the data from uncharacteristic fluctuations as shown in Fig. 9 (circles). Instead it predicts a monotonic trend in *α*/*β* for all LETs^50^. Thus, the reported RBE’s in ref. 32 corresponding to fluctuations in *α*/*β*, as shown in Fig. 8, can be interpreted as an artifact associated with numerical instabilities in conventional fitting methods. This is consistent with a simplistic cell-inactivation target theory as single-track lethal events becomes more common due to increasing LET, thus the *α*/*β* ratio increases.

## Conclusion

We have developed an improved approach to fitting clonogenic survival data for therapeutic proton and ion beams. Our approach is based on three-parameter (3D) global fitting of the high-throughput, high accuracy survival fraction data acquired after irradiation with mono-energetic proton beams. In our approach, the intrinsic correlations in cell survival data are incorporated into the fitting algorithm. As a result, background fluctuations of the extracted parameters *α* and *β* of the linear quadratic model due to uncertainties in the biological data are considerably reduced.

To demonstrate the reduction in numerical noise, we provided a comparison between traditional methods of post-processing of the clonogenic survival data and our approach. The numerical fluctuations in RBE parameters seen when an independent fitting of cell survival data is performed, can be suppressed by integrating the LET models including gMKM, LEM, and MCDS-RMF into 3D global fitting. The latter based on gMKM predicts a gradual increase in, *α*, *β*, and *α*/*β* as a function of LET, consistent qualitatively with the cell-inactivation target theory.

Further studies are on the way to implement other models such as multi-scale modeling^53, 54^ as well as MCDS-RMF into 3D global fitting of the current clonogenic survival data (that non-linear LET terms in *α* and *β* can be interpreted as higher order and more complex chromosome aberrations) as well as convolving the spectrum of lineal-energies, *dε*/*dl*, calculated by MC on *α* and *β* to take into account the effect of non-Poisson distribution. The latter is closely related to the ideas and approaches presented by Hawkins^29^ and Kase *et al*.^55^and other studies presented in refs 56–60﻿.

## Electronic supplementary material

Supplementary Materials
