# Supplementary material for: A model for relative biological effectiveness of therapeutic proton beams based on a global fit of cell survival data
Source: Sci Rep. 2017 Aug 21;7:8340. doi: 10.1038/s41598-017-08622-6 (PMC5567137; doi:10.1038/s41598-017-08622-6)
Supplement: Supplementary file 1 — Supplementary Materials [file 41598_2017_8622_MOESM1_ESM.pdf]

A model for relative biological effectiveness of therapeutic proton beams based on a global fit of cell survival data

Ramin Abolfath[1*], Christopher R. Peeler[1], Mark Newpower[1], Lawrence Bronk[2], David Grosshans[2], and Radhe Mohan[1#]

Departments of Radiation Physics[1] and Radiation Oncology[2], The University of Texas MD Anderson Cancer Center, Houston, TX 77030 USA

Corresponding Authors:
* ramin1.abolfath@gmail.com / ramin.abolfath@yale.edu
# rmohan@mdanderson.org

Supplementary Materials

Appendix – renormalization of radiobiological indices by energy loss fluctuations.
This appendix describes the steps in the mathematical derivation of Eqs. (1) to (4) and the basis of the cell survival in the generalized microdosimetric kinetic model (gMKM) introduced in this study. More specifically we provide an analytical derivation of the solutions of the RMF model and the perturbative corrections around these solutions by taking into account the statistical fluctuations in energy deposition for an arbitrary distribution function, hence the renormalization of α and β. We consider a limiting case by systematically incorporating the contribution of the higher order fluctuations to the Gaussian distribution in energy deposition to correct the dependence of β on lineal energy. Such perturbative expansion predicts an exact solution for α as a linear function of lineal energy (as known in the microdosimetry literature for decades), while β shows quadratic dependence.

As pointed out in the main text, the linear density of the atomistic excitations and ionizations undergo a transition to a highly compact distribution as the primary protons slow down. Therefore, the deviation from a Poisson distribution appears to be significant at the end of the range of proton. Figures 1A and 1B in the main text illustrate the ionization events within the cellular dimensions generated by traversing a proton with initial energy of 80 MeV and 1 MeV. The MC simulation has performed by using Geant4 DNA. As illustrated in these figures, the number of events in low energies (e.g., 1 MeV) is orders of magnitude greater than high energies (e.g., 80 MeV). Such difference in compactness of the ionization clearly justifies deviation from the Poisson distribution as the proton keeps losing kinetic energy.

Refs. [28,29,57] describes the original approach in mathematical formulation of the cell survival in the standard microdosimetric kinetic model (MKM), starting from the repair-misrepair fixation model (RMF) [49-50] in a cell nucleus domain. In this approach, Hawkins has shown that the time-integrated solution of the linearized RMF mass-action equations, averaged over the ensemble of the cell nucleus domains, leads to the linear-quadratic dependence of cell survival on the deposited dose, the first two terms in Eq.(1). Moreover this model predicts α to be a linear function of LET and β a constant and independent of LET.
A similar approach with a distinction of including the non-linear (quadratic) term in the solutions of the RMF mass-action equations that accounts for the chromosome misrepair binary end-joining leads to higher order deposited dose and LET terms. These terms in Eqs. (1) to (4) are effectively perturbative corrections to the linear expansion of α and β calculated in the MK model as presented in Ref. [57].

Cell survival in RMF and MK model: The mass-action equations describing chromosome repair-misrepair binary end-joining introduced in Refs. [49-50] and [57] are given by

$$\frac{dn}{dt} = \mu\dot{z} - \lambda n - \gamma n^2, \qquad (A1)$$
$$\frac{dN}{dt} = \lambda_L n + \gamma_L n^2. \qquad (A2)$$

Here n and N represent the number of DSBs and the lethal lesions per cell in a domain, equivalent of type II and I lesions in the MK model [57], respectively. $\dot{z}$ is the microscopic dose rate, μ is the number of DSBs per domain per Gy. λ and γ are repair and binary misrepair end-joining coefficients corresponding to DSB restitution rate and binary DSB removal rate (the average rate at which binary misrepair removes DSBs by using them in lethal lesions or in harmless rearrangement), respectively.

Due to large fluctuations of energy deposition in sub-micrometer volumes, the ionizing radiation is characterized by the probability distribution of specific energy, and single event specific and lineal energies and their expectation values, the frequency-mean (z_F, y_F), and dose-averaged (z_D, y_D), as well as their higher order statistical moments. The cell survival fraction is therefore given by

$$SF = e^{-\bar{N}}, \qquad (A3)$$

where $\bar{N}$ is the mean lethal lesions, averaged over specific energy distribution, in the ensemble of domains in all cell nuclei.

Linear solutions and LQ cell survival: First we consider the linear approximation in RMF model, Eq. $(A3)$, in which $\gamma = 0$, corresponding to Eq.(7) in Ref. [57]. In this limit, the analytical solution of Eqs. $(A1)$ and $(A2)$ can be easily found using the Green's function method

$$n_0(t) = \int_{-\infty}^{+\infty} dt'\, G_r(t - t')\dot{z}(t'). \qquad (A4)$$

Here n_0 is the solution of Eq. (A1) in the linear approximation. It is straightforward to justify that the homogeneous solution of Eq. (A1) is identical to zero, thus we do not consider it in Eq. (A4). It is also a straightforward calculation to show $G_r(t - t') = \mu e^{-\lambda(t-t')}\theta(t - t')$, where $\theta(t - t')$ is the Heavyside function, i.e., $\theta = 1$ if $t \geq t'$ and 0 otherwise. The steps in calculating retarded Green's function, G_r, include converting the integral equation, Eq. (A4), to a differential equation for G by substituting (A4) in (A1) and imposing the initial condition $\bar{n}_0 = 0$ for $t < 0$ where $\dot{z} = 0$. Similarly we define $SF_0 = e^{-\bar{N}_0}$ where $\bar{N}_0 = \int_{-\infty}^{+\infty} dt' \left[\lambda_L \bar{n}_0 + \gamma_L \overline{n_0^2}\right]$. Here the bar over N_0 denotes energy deposition averaging on the ensemble of cell nuclei domains, specific to a lineal-energy distribution.

For an acute radiation dose, $\dot{z}(t) = z\delta(t)$, the solution of Eq.(A4), $n_0(t) = \mu z e^{-\lambda t}\theta(t)$, leads to $\bar{N}_0 = \frac{\lambda_L}{\lambda}\mu\bar{z} + \frac{\gamma_L}{2\lambda}\mu^2\overline{z^2}$. By averaging over the lineal-energy distribution and all cell nuclei and their domains we obtain a linear-quadratic model in cell-survival

$$-\ln(SF) = \alpha\bar{z} + \beta\bar{z}^2, \qquad (A5)$$

where $\alpha = \frac{\lambda_L}{\lambda}\mu + \frac{\gamma_L}{2\lambda}\mu^2 z_D$ and $\beta = \frac{\gamma_L}{2\lambda}\mu^2$. Here we use the identity $\overline{z^2} = \bar{z}(\bar{z} + z_D)$ [58] that accounts for the spatial averaging of the energy deposition fluctuations, where $z_D = \frac{1}{z_F}\int_0^\infty z^2 f_1(z)dz$, $z_F = \int_0^\infty z f_1(z)\, dz$ and $f_1(z)$ is the single event distribution function of specific energy deposition, a counterpart distribution function of the lineal-energy $f(y)$. Furthermore $\bar{z} = \int_0^\infty zF(z; n_T)dz = n_T z_F$ where $F(z; n_T)$ is distribution function accounting for all events and n_T is mean number of events and/or tracks. Applying a relation between z and y (see e.g., Eq.(II.28) in Ref. [57] or Eq. (8) in Ref. [55]), $z_D = l(y_D/m)$, where $m = \rho V$ and l are the average mass and the chord length of a MKM domain, with ρ and V, the mass density and the average volume of the domains, we obtain $\alpha = \frac{\lambda_L}{\lambda}\mu + \frac{\gamma_L}{2\lambda}\mu^2 \frac{1}{\rho(V/l)} y_D$. Considering the RMF and MKM constants in α and β as phenomenological parameters we end up with two relations frequently used in the literature for fitting RBE data, (see for example Refs. [55,57])

$$\alpha = \alpha_0 + \beta \frac{1}{\rho\pi r_d^2} y_D; \quad \beta = \beta_x. \qquad (A6)$$

Here α_0, β_x, and r_d (the radius of a spherical domain) are the phenomenological fitting parameters.

Non-linear expansion of RMF solutions, going beyond LQ cell survival: We now turn to perform a perturbative expansion to calculate the non-linear solution of the RMF model, Eqs. (A1) and (A2). To go beyond the RMF linear solutions presented above, we assume γ to be a small parameter, hence we expand n about n_0 perturbatively and linearize the resulting mass-action kinetic equation to obtain the dynamics of the small fluctuations describing deviations from linear DSB solutions. We define $n_1 = n - n_0$ and recall Eq. (A1) to obtain a linear mass-action equation for n_1

$$\frac{dn_1}{dt} = -\lambda n_1 - \gamma(2n_0 n_1 + n_0^2) + O(n_1^2). \qquad (A5)$$

Here n is the exact solution of the RMF model. As we defined, n_0 is the linear solution of the RMF model, hence n_1 describes the difference between exact and linear solutions. It is more convenient to transform Eq. (A5) into a more compact form

$$\frac{dn_1}{dt} + \eta(t)n_1(t) = \xi(t), \qquad (A6)$$

where $\eta = \lambda + 2\gamma n_0$ and $\xi = \gamma n_0^2$. The solution of Eq. (A6) can be calculated exactly

$$n_1(t) = e^{-\varphi(t)} \int_{-\infty}^{t} dt' \xi(t') e^{\varphi(t')}, \qquad (A7)$$

where $\varphi(t) = \lambda t + 2\gamma \int_{-\infty}^{t} dt' n_0(t')$. Linearizing Eq. (A7) in terms of γ, assuming γ is a small parameter, leads to

$$n_1(t) = \gamma e^{-\lambda t} \int_{-\infty}^{t} dt' n_0^2(t') e^{\lambda t'} + O\left(\frac{\gamma^2}{\lambda^2}\right). \qquad (A8)$$

Substituting the linear solution calculated above, $n_0(t) = \mu z e^{-\lambda t}\theta(t)$, in Eq. (A8) yields

$$n_1(t) = \frac{\gamma}{\lambda}\mu^2 z^2\left(1 - e^{-\lambda t}\right)e^{-\lambda t}, \qquad (A9)$$

hence

$$n = n_0 + n_1 = n_0 - \frac{\gamma}{\lambda}[n_0 - \mu z]n_0 + O(n_0^3). \qquad (A10)$$

From Eq.(A10) and n_0 the cell-survival can be calculated, $-\ln(\mathrm{SF}) = \int_{-\infty}^{+\infty} dt' \left[\lambda_L \bar{n} + \gamma_L \overline{n^2}\right]$, hence

$$-\ln(\mathrm{SF}) = \frac{\lambda_L}{\lambda}\mu\bar{z} + \frac{1}{2}\left[\frac{\lambda_L}{\lambda}\frac{\gamma}{\lambda} + \frac{\gamma_L}{\lambda}\right]\mu^2\overline{z^2} + \frac{1}{3}\frac{\gamma_L}{\lambda}\frac{\gamma}{\lambda}\mu^3\overline{z^3} + \left[-\frac{\gamma_L}{6\lambda}\frac{\gamma^2}{\lambda^2}\mu^4\overline{z^4} + O\left(\frac{\gamma^2}{\lambda^2}\overline{z^4}\right)\right] + O\left(\overline{z^5}\right) \qquad (A11)$$

The last two terms in Eq. (A11) are the contribution of the terms omitted in Eq. (A8) due to linearizing n in the limit where γ is negligible. To transform (A11) to a form similar to the linear-quadratic model we must calculate the statistical fluctuations in microscopic dose deposition throughout the averaging over cell nucleus domains, assuming equivalence between the ensemble averaging over the domains and the spatial averaging of the energy deposition fluctuations over the cell nuclei. In general, these fluctuations can be recursively reduced to lower power fluctuations, namely

$$\overline{z^2} = \bar{z}^2 + z_{21}\bar{z}, \qquad (A12a)$$

$$\overline{z^3} = \bar{z}^3 + z_{32}\bar{z}^2 + z_{31}^2\,\bar{z}\,, \qquad (A12b)$$

$$\overline{z^4} = \bar{z}^4 + z_{43}\bar{z}^3 + z_{42}^2\bar{z}^2 + z_{41}^3\bar{z}\,. \qquad (A12c)$$

...

and in general

$$\overline{z^i} = \sum_{j=1}^{i} z_{ij}^{i-j}\,\bar{z}^j$$

Here z_{ij} are coefficients in the expansion with the physical dimension identical to the dimension of specific energy, Gy, and can be calculated by integrating over single event energy deposition distribution, $f_1(z)$. For example $z_{21} = z_D$, $z_{31}^2 = \frac{1}{z_F}\int_0^\infty z^3 f_1(z)dz$, $z_{32} = 3z_D$, $z_{41}^3 = \frac{1}{z_F}\int_0^\infty z^4 f_1(z)dz$, $z_{42}^2 = 3z_D^2 + 4z_{31}^2$, $z_{43} = 6z_D$, …

Applying Eqs. (A12a) to (A12c) in Eq. (A11) and keeping up to the quadratic term in macroscopic dose, $D = \bar{z}$, we find

$$-\ln(\mathrm{SF}) = \left[\frac{\lambda_L}{\lambda}\mu + \frac{1}{2}\left(\frac{\lambda_L}{\lambda}\frac{\gamma}{\lambda} + \frac{\gamma_L}{\lambda}\right)\mu^2 z_{21} + \frac{1}{3}\frac{\gamma_L}{\lambda}\frac{\gamma}{\lambda}\mu^3 z_{31}^2 + \cdots\right]\bar{z} + \left[\frac{1}{2}\left(\frac{\lambda_L}{\lambda}\frac{\gamma}{\lambda} + \frac{\gamma_L}{\lambda}\right)\mu^2 + \frac{1}{3}\frac{\gamma_L}{\lambda}\frac{\gamma}{\lambda}\mu^3 z_{32} + \cdots\right]\bar{z}^2 + \cdots \qquad (A13)$$

Hence

$$\alpha = \frac{\lambda_L}{\lambda}\mu + \frac{1}{2}\left(\frac{\lambda_L}{\lambda}\frac{\gamma}{\lambda} + \frac{\gamma_L}{\lambda}\right)\mu^2 z_{21} + \frac{1}{3}\frac{\gamma_L}{\lambda}\frac{\gamma}{\lambda}\mu^3 z_{31}^2 + \cdots \qquad (A14)$$

$$\beta = \frac{1}{2}\left(\frac{\lambda_L}{\lambda}\frac{\gamma}{\lambda} + \frac{\gamma_L}{\lambda}\right)\mu^2 + \frac{1}{3}\frac{\gamma_L}{\lambda}\frac{\gamma}{\lambda}\mu^3 z_{32} + \cdots \qquad (A15)$$

In the limit where $\gamma = 0$, Eqs. (A14) and (A15) reduce to $\alpha = \frac{\lambda_L}{\lambda}\mu + \frac{\gamma_L}{2\lambda}\mu^2 z_D$ and $\beta = \frac{\gamma_L}{2\lambda}\mu^2$, the linear MKM approximation [50]. By expanding z_{ij} around z_D in Eqs. (A14) and (A15), e.g., $\int_0^\infty z^n f_1(z)dz = \int_0^\infty (z_D + \delta z)^n f_1(z)dz = \sum_{k=0}^n \frac{n!}{k!(n-k)!} z_D^k \int_0^\infty (z - z_D)^{n-k} f_1(z)dz = \sum_{k=0}^n \frac{n!}{k!(n-k)!} z_D^k \tilde{f}_{n-k}(z_D)$ where $\delta z = z - z_D$ and $\tilde{f}_k(z_D) = \int_0^\infty (z - z_D)^k f_1(z)dz$, and using $z_{ij} = l y_{ij}/m$, and a linear relationship between y_D and L [59,60], one may find α and β to be a power series in lineal energy and LET as given by Eq.(3) and (4). The constants in Eqs. (A14) and (A15) can be determined phenomenologically by fitting α and β to the experimental data as illustrated in the text, i.e., $\alpha = \sum_{k=1}^n b_{k,1} L^{k-1}$, and $\beta = \sum_{k=1}^{n-1} b_{k+1,2} L^{k-1}$. As seen in these equations, the contribution from the energy loss fluctuations renormalizes the LQ biological parameters α and β to infinite orders in z_D and y_D.

Gaussian fluctuations

It is interesting to calculate z_{ij}, α and β for a widely used Gaussian distributed function as in the limit $\bar{z} \gg 0$, the Poisson distribution can be approximated to a Gaussian (central limit theorem) with variance $\sigma^2 = z_{GD}\bar{z}_G$

$$F(z; n_T) \longrightarrow F_G(z; n_T) = \frac{1}{\sigma\sqrt{2\pi}} \exp\left(-\frac{(z - \bar{z}_G)^2}{2\sigma^2}\right)$$

Here we change the notations $\longrightarrow F_G$, $\bar{z} \longrightarrow \bar{z}_G$, $z_F \longrightarrow z_{GF}$ and $z_D \longrightarrow z_{GD}$ with the subscript G to denote the averaging over the Gaussian distribution function. It is straightforward calculation to find the Gaussian version of equations (A12a – A12c)

$$\overline{z^2} \longrightarrow \overline{z_G^2} = \bar{z}_G^2 + z_{GD}\bar{z}_G\,, \qquad (A12d)$$

$$\overline{z^3} \longrightarrow \overline{z_G^3} = \bar{z}_G^3 + 3z_{GD}\bar{z}_G^2, \qquad (A12e)$$

$$\overline{z^4} \longrightarrow \overline{z_G^4} = \bar{z}_G^4 + 6z_{GD}\bar{z}_G^3 + 3z_{GD}^2\bar{z}_G^2, \qquad (A12f)$$

$$\overline{z^5} \longrightarrow \overline{z_G^5} = \bar{z}_G^5 + 10z_{GD}\bar{z}_G^4 + 15z_{GD}^2\bar{z}_G^3, \qquad (A12g)$$

$$\overline{z^6} \longrightarrow \overline{z_G^6} = \bar{z}_G^6 + 15z_{GD}\bar{z}_G^5 + 45z_{GD}^2\bar{z}_G^4 + 15z_{GD}^3\bar{z}_G^3, \qquad (A12h)$$

...

where all statistical fluctuations and higher order moments are reduced to two variables $z_{GD} = \frac{1}{z_{GF}}\int_0^\infty z^2 f_{G1}(z)dz$ and $\bar{z}_G = n_T z_{GF}$ where $z_{GF} = \int_0^\infty z f_{G1}(z)\,dz$. A comparison between Eqs. (A12d – A12f) and (A12a – A12c) shows that $z_{G,21} = z_D, z_{G,32} = 3z_D, z_{G,31} = 0, z_{G,43} = 6z_D, z_{G,42} = \sqrt{3}z_D, z_{G,41} = 0, \ldots$ Note that the fluctuations such as $z_{G,31}$, $z_{G,32}$, $z_{G,41}$ are negligible because of specific symmetry of $G(z)$.

Similar to a general energy loss fluctuations, the contributions of the Gaussian fluctuations in energy deposition introduce correction factors to α and β. Because the contribution to $\bar{z}_G$ from $\overline{z_G^3}$ and beyond are identical to zero, there is no correction beyond the linear term in z_{GD} and hence in y_{GD}. This is evident from Eq. $(A12e)$ as the lowest order correction to specific energy and deposition dose is quadratic. Hence the Gaussian model predicts α to be only linear dependence on lineal energy and LET

$$\alpha_G = \frac{\lambda_L}{\lambda}\mu + \frac{1}{2}\left(\frac{\lambda_L}{\lambda}\frac{\gamma}{\lambda} + \frac{\gamma_L}{\lambda}\right)\mu^2 z_{GD}. \qquad (A16)$$

Similarly for β the corrections beyond $\overline{z^5}$ fluctuations vanish exactly as the lowest order correction to the dose in Eq. $(A12g)$ is cubic. Hence we find a closed form for β

$$\beta_G = \frac{1}{2}\left(\frac{\lambda_L}{\lambda}\frac{\gamma}{\lambda} + \frac{\gamma_L}{\lambda}\right)\mu^2 + \frac{\gamma_L}{\lambda}\frac{\gamma}{\lambda}\mu^3 z_{GD} + \left(-\frac{\gamma_L}{6\lambda}\frac{\gamma^2}{\lambda^2} + O\left(\frac{\gamma^2}{\mu^4\lambda^2}\right)\right)3\mu^4 z_{GD}^2. \qquad (A17)$$

As seen in these equations, the contribution from the fluctuations in Gaussian model of energy deposition correct the LQ biological parameters α_G and β_G to scale linearly and quadratically in z_D and y_D. We note that the radiobiological models that start from the equations equivalent of $(A16)$ and $(A17)$ are implicitly assuming a Gaussian-type symmetry in the energy-loss processes. This includes models with linear dependence on LET in α and constant β (e.g., by neglecting higher order μ terms in α and β beyond quadratic terms). Such models neglect the importance of asymmetries hidden in more realistic distribution functions such as the Landau [45] and/or Vavilov [46] distribution functions, responsible for observed nonlinearities in biological responses.

Neyman's distribution of type A and DSB distribution

We devote this section to illustrate the effect of deviation from Poisson distribution on statistical fluctuations of specific energy, DSB distribution, and biological indices α and β. Specifically we start with construction of the Neyman's distribution function from first principles for DSB induction processes and calculate the higher order moments in specific energy needed for incorporation of the repair and misrepair mechanisms to fit globally the cell survival data.

We consider the normalized distribution function to describe stochastic energy deposition in DNA material [58,59] as given below

$$F(z;\bar{\nu}) = \sum_{\nu=0}^{\infty} p_\nu(\bar{\nu})\, f_\nu(z), \qquad (A18)$$

where $p_\nu(\bar{\nu}) = (\bar{\nu}^\nu/\nu!)\exp(-\bar{\nu})$ describes the Poisson distributed events in an ensemble of single tracks. Here $\bar{\nu}$ denotes the average number of energy deposition events and $f_\nu(z)$ is the distribution of specific energy imparted from passage of a single track within z and $z + dz$ resulted from exactly ν energy deposition events. The stochastic process as such is sketched in Fig. (1A).

We denote ε_ν the deposited energy resulted from exactly ν events in mass m of DNA material corresponding to specific energy $z = \frac{1}{m}\sum_{i=1}^{\nu}\varepsilon_i = \nu\bar{\varepsilon_\nu}/m$ where $\bar{\varepsilon_\nu} = \frac{1}{\nu}\sum_{i=1}^{\nu}\varepsilon_i$. The occurrence of k DSBs resulted from energy deposition requires balance in energy transfer to DNA. More specifically $\nu\bar{\varepsilon_\nu} = \nu\sum_{i=1}^{\Delta}\epsilon_i = k\bar{\epsilon}_\Delta$ where $\bar{\epsilon}_\Delta = \frac{1}{\Delta}\sum_{i=1}^{\Delta}\epsilon_i$ is the typical energy results in breaking chemical bonds for induction of a single DSB and $k = \nu\Delta = 0,1,2,\ldots$ counts number of DSBs. Δ is the number of induced DSBs in an event. Hence the energy balance in an exactly ν events processes requires $z = \bar{\epsilon}_\Delta\nu\Delta/m$. Further simplification can be performed by averaging over DSB population per event subsequently by averaging over events that yields $\bar{\bar{k}} = \overline{\nu\bar{\Delta}} = \bar{\nu}\bar{\Delta}$. Here $\bar{\Delta}$ is average number of DSBs per event, independent of ν. The double bars over k denotes two independent averagings; hence the order of averaging is not an issue. Also note that $n = \bar{\bar{k}}$ and $\mu = m/\bar{\epsilon}_\Delta$ in Eq.(A1) hence $\dot{z} = (\bar{\Delta}/\mu)\dot{\bar{\nu}}$.

The distribution of DSBs in a class of events, specified by given ν, can be uniquely determined by the corresponding energy deposition distribution. The DSB partition function, $Q_k(\bar{\Delta};\bar{\nu})$, the

probability distribution in finding exactly k DSBs, can be calculated from Eq.(A18) where $f_\nu(z) = \sum_{k=0}^{\infty} p_k(z)\delta(z-\nu\bar{\Delta}/\mu)$. The insertion of δ-function, the DSB density of states, enforces a constraint on the energy transfer balance resembling Fermi golden rule formulation of transition rates and the perturbation theory in quantum physics. Substituting $f_\nu(z)$ in Eq.(A18) and integrating over z yields

$$1 = \int_0^\infty F(z;\bar{\nu})\,dz = \sum_{\nu=0}^{\infty} p_\nu(\bar{\nu}) \int_0^\infty \sum_{k=0}^{\infty} p_k(z)\delta(z-\nu\bar{\Delta}/\mu)\,dz$$

$$= \sum_{k=0}^{\infty}\sum_{\nu=0}^{\infty} p_\nu(\bar{\nu})p_k(\nu\bar{\Delta}) = \sum_{k=0}^{\infty} Q_k(\bar{\Delta};\bar{\nu}), \qquad (A19)$$

Thus

$$Q_k(\bar{\Delta};\bar{\nu}) = \sum_{\nu=0}^{\infty} p_\nu(\bar{\nu})p_k(\nu\bar{\Delta}). \qquad (A20)$$

Further approximation can be performed by considering Poisson distribution for DSB events, i.e., $p_k(\nu\bar{\Delta}) = (\nu\bar{\Delta})^k/k!\,\exp(-\nu\bar{\Delta})$ that reduces $Q_k(\bar{\Delta};\bar{\nu})$ in Eq.(A20) to Neyman's distribution of Type A (see for example Refs. [47,48]). Accordingly, the probability in finding a DNA with zero DSB is given by $Q_0 = \exp(-\bar{\nu}(1-\exp(-\bar{\Delta}))) \approx \exp(-\bar{\nu}\bar{\Delta})$. In this equation, $\bar{\nu}\bar{\Delta}$ is the average number of events times the average number of DSBs per event.

From Eq. (A20), it is now straightforward to calculate the statistical moments of DSBs and the corrections to biological indices α and β by inclusion of DSB fluctuations, as discussed in the main text. Here we systematically show that the DSB partition function given by $Q_k(\bar{\Delta};\bar{\nu})$ in Eq. (A20) provides all statistical moments we need for this analysis. For clarification of notations we first check the self-consistency of equations by calculating the first moment

$$\bar{\bar{k}} = \sum_{k=0}^{\infty} kQ_k(\bar{\Delta};\bar{\nu}) = \bar{\nu}\bar{\Delta}.$$

Furthermore

$$\overline{\overline{k(k-1)}} = \sum_{k=0}^{\infty} k(k-1)Q_k(\bar{\Delta};\bar{\nu}) = (\bar{\nu}\bar{\Delta})^2 + (\bar{\nu}\bar{\Delta})\bar{\Delta} = \bar{\bar{k}}^2 + \bar{\bar{k}}\bar{\Delta},$$

$$\overline{\overline{k(k-1)(k-2)}} = \sum_{k=0}^{\infty} k(k-1)(k-2)Q_k(\bar{\Delta};\bar{\nu}) = \bar{\bar{k}}^3 + 3\bar{\bar{k}}^2\bar{\Delta} + \bar{\bar{k}}\bar{\Delta}^2,$$

and in general

$$\overline{\overline{k(k-1)(k-2)\ldots(k-r)}} = \sum_{s=0}^{r} c_s \bar{\bar{k}}^{r-s+1}\bar{\Delta}^s. \qquad (A21)$$

Here c_s are the coefficients of expansion and $c_0 = 1$. By further expansion of Eq. (A21) we find a power law series dependence of DSB fluctuations on Δ that we need for the expansion of Eqs. (A1) and (A2)

$$\overline{\overline{k^r}} = \bar{\bar{k}}^r + (a_{01} + a_{11}\bar{\Delta})\bar{\bar{k}}^{r-1} + (a_{02} + a_{12}\bar{\Delta} + a_{22}\bar{\Delta}^2)\bar{\bar{k}}^{r-2} + \cdots$$

$$+ (a_{0r} + a_{1r}\bar{\Delta} + a_{2r}\bar{\Delta}^2 + \cdots + a_{rr}\bar{\Delta}^r)\bar{\bar{k}}. \qquad (A22)$$

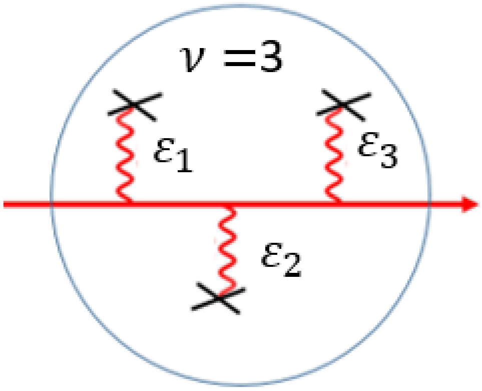

Figure 1A. Schematically shown the energy deposition of ν =3 events resulted from passage of a single track in a cell domain.

Finally we show that $\bar{\Delta} = \mu z_D$. This identity is the reminiscent of fluctuation-dissipation theorem and can be derived in the following steps

$$\frac{\overline{z^2}}{\bar{z}} = \frac{\int_0^\infty z^2\, F(z;\bar{\nu})dz}{\int_0^\infty z\, F(z;\bar{\nu})dz} = \frac{\sum_{\nu=0}^\infty p_\nu(\bar{\nu}) \int_0^\infty z^2 dz \sum_{k=0}^\infty p_k(z)\delta\left(z - \frac{\nu\bar{\Delta}}{\mu}\right)}{\sum_{\nu=0}^\infty p_\nu(\bar{\nu}) \int_0^\infty z\, dz \sum_{k=0}^\infty p_k(z)\delta\left(z - \frac{\nu\bar{\Delta}}{\mu}\right)}.$$

After integrating over δ-function we find

$$\frac{\overline{z^2}}{\bar{z}} = \frac{\sum_{k=0}^\infty \sum_{\nu=0}^\infty p_\nu(\bar{\nu}) p_k(\nu\bar{\Delta})(\nu\bar{\Delta}/\mu)^2}{\sum_{k=0}^\infty \sum_{\nu=0}^\infty p_\nu(\bar{\nu}) p_k(\nu\bar{\Delta})\,(\nu\bar{\Delta}/\mu)} = \frac{\bar{\Delta}}{\mu} \frac{\sum_{\nu=0}^\infty \nu^2 p_\nu(\bar{\nu}) \sum_{k=0}^\infty p_k(\nu\bar{\Delta})}{\sum_{\nu=0}^\infty \nu p_\nu(\bar{\nu}) \sum_{k=0}^\infty p_k(\nu\bar{\Delta})}.$$

Because $\sum_{k=0}^\infty p_k(\nu\bar{\Delta}) = 1$ and $\sum_{\nu=0}^\infty \nu^2 p_\nu(\bar{\nu}) = \overline{\nu^2} = \bar{\nu}(\bar{\nu}+1)$ and $\sum_{\nu=0}^\infty \nu p_\nu(\bar{\nu}) = \bar{\nu}$ we find

$$\frac{\overline{z^2}}{\bar{z}} = \frac{\bar{\Delta}}{\mu}(\bar{\nu}+1),$$

hence $\bar{\Delta} = [\mu/(\bar{\nu}+1)](\overline{z^2}/\bar{z})$. For a class of single events, $\overline{z^2}/\bar{z}$ reduces to z_D. More specifically

$$z_D = \left.\frac{\overline{z^2}}{\bar{z}}\right|_{\nu=1} = \left.\frac{\int_0^\infty z^2\, F(z;\bar{\nu})dz}{\int_0^\infty z\, F(z;\bar{\nu})dz}\right|_{\nu=1} = \frac{\sum_{\nu=0}^\infty p_\nu(\bar{\nu}) \int_0^\infty z^2 dz \sum_{k=0}^\infty p_k(z)\delta\left(z - \frac{\nu\bar{\Delta}}{\mu}\right)\delta_{\nu,1}}{\sum_{\nu=0}^\infty p_\nu(\bar{\nu}) \int_0^\infty z\, dz \sum_{k=0}^\infty p_k(z)\delta\left(z - \frac{\nu\bar{\Delta}}{\mu}\right)\delta_{\nu,1}}.$$

Similarly after integrating over δ-function we find

$$z_D = \frac{\sum_{k=0}^\infty \sum_{\nu=0}^\infty p_\nu(\bar{\nu}) p_k(\nu\bar{\Delta})(\nu\bar{\Delta}/\mu)^2 \delta_{\nu,1}}{\sum_{k=0}^\infty \sum_{\nu=0}^\infty p_\nu(\bar{\nu}) p_k(\nu\bar{\Delta})\,(\nu\bar{\Delta}/\mu)\delta_{\nu,1}} = \frac{\bar{\Delta}}{\mu} \frac{\sum_{\nu=0}^\infty \nu^2 p_\nu(\bar{\nu}) \sum_{k=0}^\infty p_k(\nu\bar{\Delta})\delta_{\nu,1}}{\sum_{\nu=0}^\infty \nu p_\nu(\bar{\nu}) \sum_{k=0}^\infty p_k(\nu\bar{\Delta})\,\delta_{\nu,1}} = \frac{\bar{\Delta}}{\mu}.$$

Combining the results of these equations into Eq.(A22) and using transformation, $k \longrightarrow \mu z$ in Eq.(A22) and multiplying that equation by μ^{-r} and substituting $\bar{\Delta} = \mu z_D$, we obtain

$$\overline{z^r} = \bar{z}^r + (b_{01} + b_{11} z_D)\bar{z}^{r-1} + (b_{02} + b_{12} z_D + b_{22} z_D^2)\bar{z}^{r-2} + \cdots + (b_{0r} + b_{1r} z_D + b_{2r} z_D^2 + \cdots + b_{rr} z_D^r)\bar{z}. \qquad (A32)$$

Here $b_{sr} = a_{sr}\mu^{s-r}$ are the expansion coefficients. In numerical fitting procedure to the experimental cell-survival data we consider these coefficients as phenomenological adjustable parameters. Recalling $z_D = l(y_D/m)$ and following the derivation steps presented in previous section, we arrive at similar expressions for α and β and the power series dependences on lineal energy.

The results given in Eqs. (A19-A22) for Neyman's distribution is in contrast with the Poisson distribution that only leads to the first term in the expansion given by Eq. $(A21)$ and (A22)

$$\overline{\overline{k(k-1)(k-2)\ldots(k-r)}} = \bar{\bar{k}}^r \qquad (A23)$$

Hence

$$\overline{\overline{k^r}} = \bar{\bar{k}}^r + a_{01}\bar{\bar{k}}^{r-1} + a_{02}\bar{\bar{k}}^{r-2} + \cdots + a_{0r}\bar{\bar{k}}, \qquad (A24)$$

with no dependence on Δ and lineal energy in Eq. (A24). Thus

$$\overline{z^r} = \bar{z}^r + b_{01}\bar{z}^{r-1} + b_{02}\bar{z}^{r-2} + \cdots + b_{0r}\bar{z}. \qquad (A25)$$

Substituting these results into Eqs. (A12a-c), it is now straightforward to show that the Poisson distribution leads to no dependence of α and β on lineal energy and LET consistent with the results presented by Sachs *et al.* [50]. In contrast the Neyman's distribution leads to a power law dependence of α and β on lineal energy and LET as discussed in the main text. The use of Neyman's distribution in RMF-MCDS model reported by Carlson *et al.* in Ref. [35] where z_F and LET are resulted linearly in α where β shows no dependence. One difference between derivation presented in our current study and the one presented in Ref. [35] is the incorporation of fluctuations that supersede z_D, instead of z_F in α and β such that the formulation of our linear model shows consistency with Hawkins' MK linear models [28,29,57].
